# Voriconazole Induced Cutaneous Squamous Cell Carcinoma in an Immunocompetent Patient

**DOI:** 10.7759/cureus.25508

**Published:** 2022-05-30

**Authors:** Kevin Parza, Pratishtha Singh, Jessica Cvinar, Terence Zimmerman, Brian Watson, Mohamed Faris

**Affiliations:** 1 Internal Medicine, Grand Strand Medical Center, Myrtle Beach, USA; 2 Otolaryngology, Grand Strand Medical Center, Myrtle Beach, USA; 3 Pathology, Grand Strand Medical Center, Myrtle Beach, USA

**Keywords:** photosensitivity reaction, radiation therapy, parotid mass, mass, pulmonary aspergillosis, voriconazole, cutaneous squamous cell carcinoma

## Abstract

Voriconazole therapy can be associated with hair loss, vision changes, and skin phototoxicity, but rarely is it associated with the development of skin cancer. We present a case of an immunocompetent 42-year-old Caucasian male with a past medical history significant for chronic pulmonary aspergillosis (CPA) and prior cutaneous squamous cell carcinoma (cSCC) of the left hand who arrived at our clinic for evaluation of an enlarging, non-tender left preauricular mass over the past six months. He had diffuse actinic changes and appeared older relative to his age. He had a fair complexion but was compliant with sun protection measures and minimized unnecessary ultraviolet (UV) light exposure. His left-sided facial mass was excised, and the final pathology was consistent with cSCC. His only home medication was oral voriconazole 200 mg once daily for six years for pulmonary aspergillosis. He was negative for human immunodeficiency virus (HIV) and had no history of prior transplant operations. This case highlights the importance of recognizing voriconazole as an independent risk factor in the development of cSCC, especially in patients on chronic therapy for aspergillosis.

## Introduction

Voriconazole (VRZ) and itraconazole (ITZ) are typical first-line antifungal agents utilized in the treatment of chronic aspergillosis [1]. However, multiple reports have demonstrated an increased rate of ITZ resistance with a growing preference for the usage of VRZ [2].

VRZ is a triazole antifungal that interferes with fungal cytochrome P450 activity decreasing ergosterol synthesis and inhibiting fungal cell membrane formation. It is widely used as a prophylactic antifungal medication [3] and for the treatment of invasive fungal infections. A study published by Hamandi et al. in October 2016 looked at the incidence rate of cutaneous squamous cell carcinoma (cSCC) in patients on prophylactic VRZ following lung transplant and initiation of immunosuppressive therapy. They found that there was an increased incidence of cSCC in patients with exposure to VRZ alone (adjusted hazard ratio: 2.39, 95% confidence interval: 1.31-4.37), which was statistically significant [4]. Despite the numerous studies looking at the incidence of VRZ-induced cSCC in immunocompromised patients, there have been no studies to date that determine the incidence of cSCC in immunocompetent patients such as those on VRZ for pulmonary aspergillosis.

VRZ-induced drug adverse effects can most often present as visual disturbances, but rarely this medication has been associated with skin phototoxicity [5]. We present a case of VRZ-induced cSCC in an immunocompetent patient.

## Case presentation

A 42-year-old Caucasian male with a history of chronic aspergillosis infection and cSCC of the left dorsal hand was evaluated in the clinic for enlarging left preauricular mass over the course of six months. The patient denied any associated facial pain, paresthesias, facial paralysis, or prior history of head and neck cutaneous malignancies. One year prior, he was diagnosed with a cSCC lesion on his left hand and underwent a wide excision resulting in the successful removal of all cancerous margins. The patient was Fitzpatrick I skin type but was compliant with sun protection and minimized unnecessary UV exposure. His only reported home medication was VRZ 200 mg once daily for six years for chronic pulmonary aspergillosis (CPA). Of note, the patient was immunocompetent with a negative human immunodeficiency virus (HIV) screen and had no prior pulmonary risk factors for aspergillosis. 

**Figure 1:**
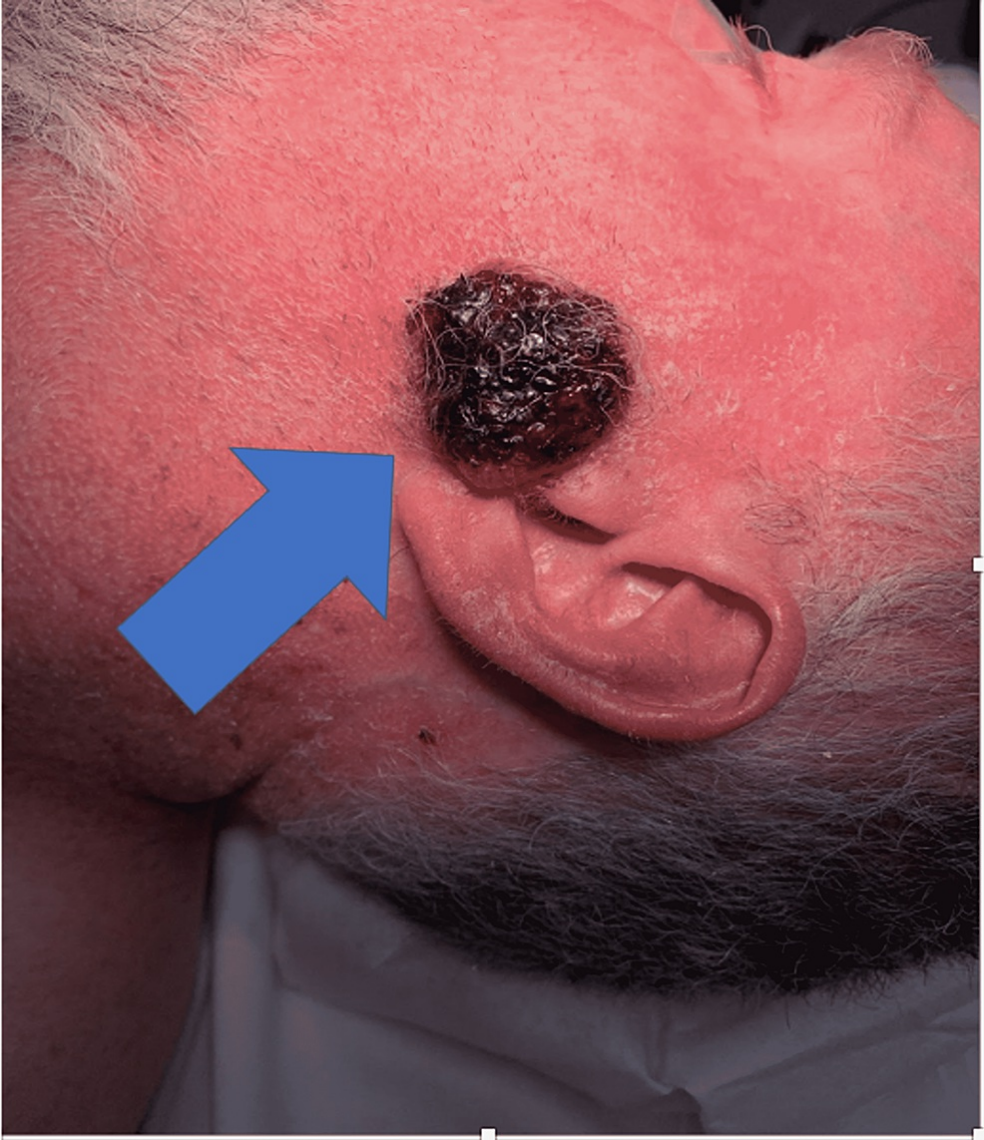
Preoperative enlarged left preauricular parotid mass (blue arrow)

**Figure 2:**
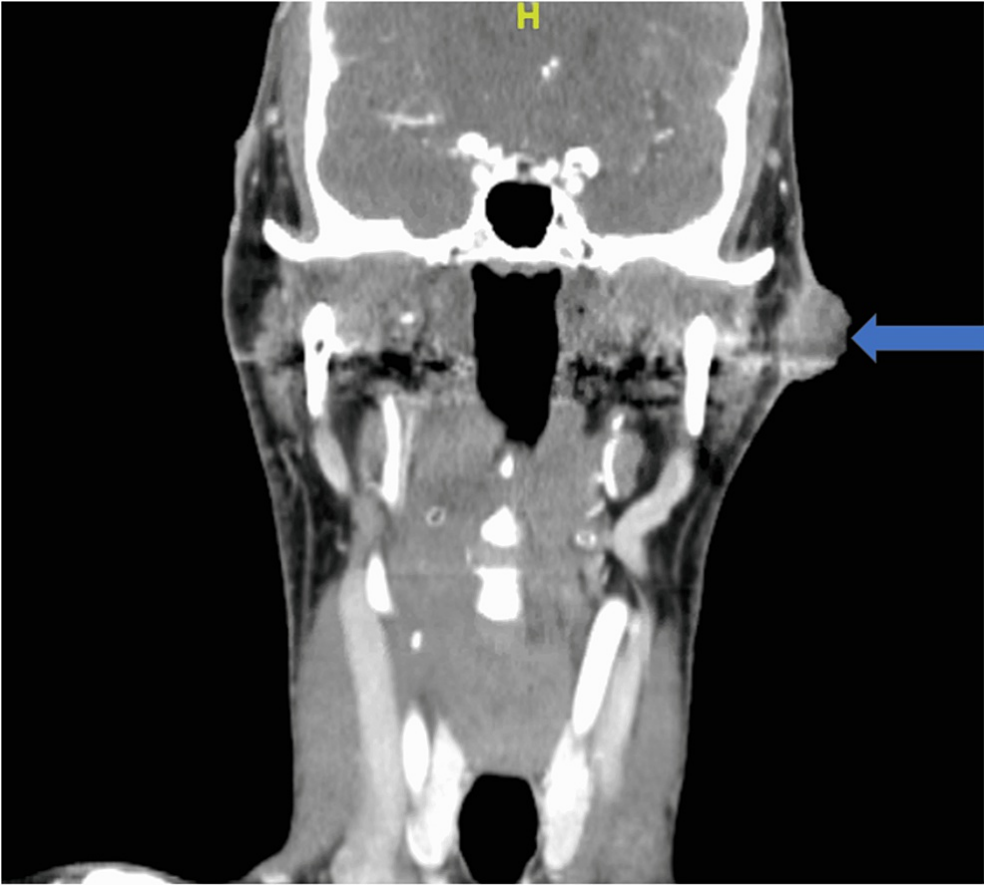
CT of the left neck (coronal view) demonstrates 3.2 x 1.9 x 2.5 cm fungating mass with concerns for malignancy (blue arrow)

**Figure 3:**
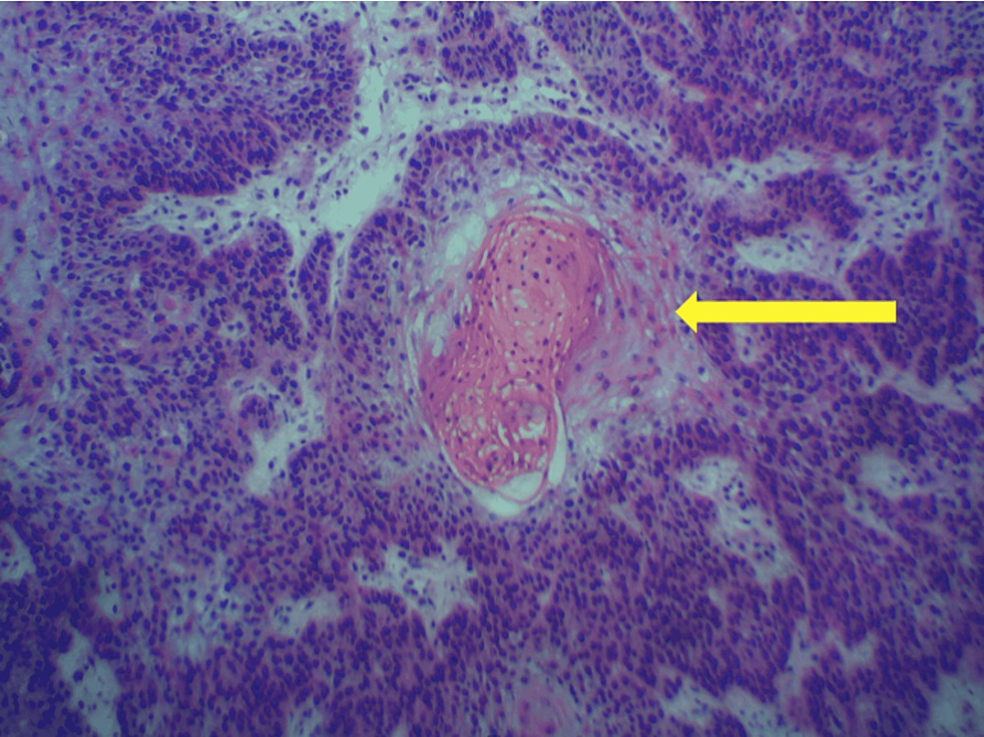
Tissue biopsy demonstrating keratin pearl (yellow arrow) consistent with cutaneous squamous cell carcinoma Hematoxylin and eosin, x100

He was hemodynamically stable. His only pertinent laboratory result was serum eosinophilia with an elevated value of 535 (cell/uL). Compared to six years prior, eosinophil levels were within normal limits. On physical examination, he had accelerated greying of his hair, appearing much older than his age. In addition, the patient had diffuse actinic changes throughout the skin. The left-sided facial mass was a raised, 3.2 cm circumferential, exophytic, fungating mass in the preauricular area. It was non-tender to touch and violaceous in color (Figure 1). He was referred for surgical management by an otolaryngologist. Computed tomography (CT) with a contrast of his head and neck demonstrated no extension into the fascial plane (Figure 2). Intraoperative frozen section pathology demonstrated cutaneous squamous cell carcinoma (Figure 3). There was no metastatic involvement of the parotid or cervical lymph nodes. Surgical management involved wide local excision of the cutaneous mass, left superficial parotidectomy, left elective level II neck dissection, and advancement flap reconstruction of the surgical defect. His postoperative course was unremarkable. Given the recurrence of cSCC, his infectious disease doctor discontinued the VRZ. In addition, he was referred to an oncologist for routine surveillance. Three months later, the patient was without CPA exacerbation and had almost near resolution of his diffuse actinic skin changes. In addition, his serum eosinophils levels returned to normal limits. 

## Discussion

Clinical as well as dermoscopic findings can strongly suggest a diagnosis of cSCC. However, a histopathologic examination is necessary to confirm the diagnosis. This can also be useful for the assessment of perineural invasion, tumor differentiation, and tumor depth, factors that are important for staging and prognosis. 

There have been case reports describing skin cancers associated with VRZ exposure, and despite being rare, it is now an established independent risk factor for the development of cutaneous malignancy. VRZ has been associated with dermatological complications; however, most are mild skin rashes consisting of non-tender macular erythema. Severe reactions can include Stevens-Johnson syndrome and toxic epidermal necrolysis [6]. Case reports have described patients developing severe photosensitivity with VRZ resulting in multifocal squamous cell carcinomas (SCCs) [7]. There have also been retrospective studies looking at VRZ-associated malignancy in transplant patients, but few studies have examined the risk to non-transplant recipient patients. 

VRZ-associated malignancy appears to be a relatively recent phenomenon, with no cases of multifocal or highly invasive SCCs complicating VRZ-induced photosensitivity reactions reported prior to 14 years ago. The exact mechanism of VRZ-induced skin cancer is still unknown. However, it is proposed that the mechanism may involve VRZ's primary metabolite, VRZ N-oxide, or, alternatively, an indirect retinoid effect of the VRZ therapy [8]. 

According to another recent retrospective cohort study conducted by Mansh et al., VRZ exposure was noted to be associated with a 73% increased risk of developing SCC, with each additional 30-day exposure at the standard dose increases the risk by 3% [9]. In addition, a retrospective study found that in immunocompromised patients, such as transplant recipients on immunosuppressants, VRZ-induced skin cancer can occur as early as six months [10]. Currently, there are no studies that measure the time of onset of VRZ use to skin cancer diagnosis in immunocompetent patients.

Environmental, genetic factors, as well as immunosuppression, can contribute to the development of cutaneous SCC. Environmental factors include, but are not limited to, ultraviolet radiation, ionizing radiation, arsenic exposure, and radon. Some genetic risk factors include a patient's family history, inherited disorders such as xeroderma pigmentosum, albinism, and other genetic syndromes. Chronic immunosuppression secondary to solid organ transplant, HIV infection, or long-term steroid use also increased the incidence of cSCC. Other causes can include smoking, human papillomavirus (HPV) infection, thiazide diuretics, and other photosensitizing drugs.

A notable risk factor relative to VRZ-induced cSCC is individuals with certain cytochrome P450 enzymes. People with poor metabolism polymorphisms of homozygous CYP219 have serum voriconazole levels exponentially higher than those without the enzyme discovered in a recent study [11]. As a result, dosage adjustment to the consumption of VRZ may need to be addressed. 

Currently, there are no accepted serum VRZ levels that correlate with the increased risk of phototoxicity secondary to VRZ use. However, there are reported cases that suggest a possible correlation between serum eosinophil levels and phototoxicity. A study by Grzegorczyk et al. evaluated the relationship between VRZ-induced colitis and eosinophilia, which demonstrated a complete resolution of symptoms and normal eosinophil count following cessation of the antifungal agent. [12]. In another case report by Vishnubhotla et al., patients initially presenting with fever of unknown origin and elevated eosinophils have an improvement in symptoms after stopping the antifungal agent [13].

Per the National Comprehensive Cancer Network (NCCN) guidelines for SCC, localized low-risk cSCC with low-risk pathologic factors, patients are often started on curettage and electrodesiccation and/or cryotherapy [14]. For most localized, low-risk cSCC, surgical excision with or without sentinel node biopsy is considered first-line therapy with adjuvant radiation therapy (RT) reserved for subsequently discovered high-risk pathologic features [15]. For individuals who are not operative candidates or who decline surgical therapy, primary radiation therapy with or without systemic therapy is considered the second line. Rowe et al. performed a meta-analysis that demonstrated that there was a five-year recurrence rate of 6.7% and 10% following definitive RT for primary and recurrent cSCC, respectively [16]. High-risk cSCC with positive surgical margins or non-surgical candidates may benefit from RT plus systemic therapy, such as programmed cell death-1 (PD-1) inhibitors and/or cetuximab [15].

## Conclusions

While common risk factors for cSCC include HIV, hematologic malignancies, and immunosuppressive medications, VRZ should not be overlooked. Clinicians caring for patients with a variety of medical conditions on VRZ therapy should consider routine dermatologic physical exams and obtain routine eosinophil levels. Cessation of the antifungal therapy may be warranted following the diagnosis of cSCC while on VRZ. However, an alternative antifungal agent should be considered in patients with CPA exacerbations and in individuals with a high risk for developing cSCC.
